# Home treatment teams and facilitated discharge from psychiatric hospital

**DOI:** 10.1017/S2045796014000304

**Published:** 2014-06-30

**Authors:** A. D. Tulloch, M. R. Khondoker, G. Thornicroft, A. S. David

**Affiliations:** King's College London, King's Health Partners, Institute of Psychiatry, London, UK

**Keywords:** Community mental health services, home care services, length of stay, mental disorders

## Abstract

**Aims.:**

There has been little research into the facilitated discharge (FD) function of Home Treatment Teams (HTTs). We aimed to explore and describe the prevalence and associations of FD and to estimate its effects on bed days during the index admission (length of stay corrected for ward leave) and on readmission.

**Methods.:**

Descriptive and regression analyses of data collected by South London and Maudsley NHS Foundation Trust on discharges from its general psychiatric wards, with multiple imputation of missing covariate values.

**Results.:**

Overall, 29% of our sample of 7891 hospital admissions involved a FD. FD was associated with female gender, diagnosis of a severe mental illness, previous home treatment, having a longer previous admission, neither being discharged to a new address nor to a care home, having no other community team and having HoNOS item scores consistent with an active depressive or psychotic mental illness. In the regression analysis, FD was associated with 4.0 fewer bed days (95% confidence interval −6.7 to −1.3; *p* = 0.0004). There was no effect on readmission.

**Conclusions.:**

Our analysis provides some support for the effectiveness of FD in slightly reducing the time spent in hospital and suggests that this may be achieved without increasing the rate of readmission. Further studies in this area are important, especially given existing research that suggests that the introduction of HTTs in England and Wales was associated with little or no change in service utilisation.

## Introduction

Since 2001, the health service in England and Wales has established a network of community-based Home Treatment Teams (HTTs), initially structured according to the Department of Health's Mental Health Policy Implementation Guide (Department of Health, 2001). HTTs – which are also known as Crisis Resolution Teams – were introduced in order to increase the capacity of community mental health services to treat people with the most acute and severe forms of depression, schizophrenia and bipolar disorder. Accordingly, the only factor that has been found to be consistently associated with the use of home treatment in published studies is having a diagnosis of a severe mental illness (Cotton *et al.* 2007). The Department of Health Mental Health Policy Implementation Guide outlines two main functions of HTTs. The first is to provide an alternative to hospital admission. The second is to enable earlier discharge from hospital by being ‘actively involved in discharge planning’ and by providing ‘intensive care at home’.

Studies of the overall effectiveness of HTTs have been reviewed elsewhere (Hubbeling & Bertram, 2012; Carpenter *et al.* 2013; Johnson, 2013). There are three studies based on individual-level data: quasi-experimental evaluations in South Islington (Johnson *et al.* 2005*a*) and Northeast Birmingham (Ford *et al.* 2001), and a randomised controlled trial in North Islington (Johnson *et al.* 2005*b*), all of which showed reductions in service use including the probability of admission. Studies based on aggregated data are less positive. Both Glover *et al.* (2006) and Jacobs & Barrenho (2011) analysed a set of national routine data, aggregated to the level of 229 Primary Care Trusts (PCTs) and linked to data on the dates at which HTTs were introduced. Glover *et al.* (2006) compared the earliest year prior to HTT introduction for which data were available with the latest year after HTT introduction for which data were available (*N* = 454). Jacobs & Barrenho (2011) used admission numbers for an average of 6 years per PCT (*N* = 1274) and modelled differences from trend at the time of the introduction of an HTT – a more powerful technique, which accounts better for the secular trend towards reducing admissions and differences between PCTs with and without HTTs. These analyses suggest that the effect of HTTs on area-level admission rates has been either fairly small (Glover *et al.* 2006) or non-existent (Jacobs & Barrenho, 2011), and furthermore that HTTs appear not to have produced any reduction in total bed days (Glover *et al.* 2006). Smaller single or dual area-level studies based on aggregated data probably add little to these large studies, and suffer from methodological problems such as poor estimation of trends before or after the introduction of an HTT (Forbes *et al.* 2010; Tyrer *et al.* 2010; Barker *et al.* 2011), or the use of a before–after design when a reducing trend in reduction in admissions appears to predate the introduction of home treatment (Jethwa *et al.* 2007), although they have shown a mixture of positive and negative findings.

Our concern in this paper is with the second, ‘facilitated discharge’ (FD) function of HTTs. In our analyses, we define receiving an FD as *beginning to be treated by an HTT during a period of admission to hospital or at its conclusion*. FD has largely been eclipsed in the literature: no published study describes its contribution to the overall activity of HTTs; no study has described the proportion of discharges that are facilitated or the characteristics of those who are treated with FD; and when HTT outcomes have been studied, only in some cases have FD patients been explicitly included (Johnson *et al.* 2005*b*).

Our aims were fourfold:

1. To document the proportion of all home treatment episodes those are FDs.
2. To explore the variables associated with being treated with FD.
3. To test the hypothesis that FD would reduce the number of bed days within the admission (length of stay – LOS – minus any leave days).
4. To test the hypothesis that FD would reduce the rate of readmission.

We studied the HTTs operated by South London and Maudsley NHS Foundation Trust (‘the Trust’), which together serve four London Boroughs. Croydon had a single team throughout the study period, comprising approximately 16 full-time staff. The three other boroughs had two or three teams per borough at the start of the study period, merging into a single team from December 2006 in Lambeth, from November 2010 in Lewisham and from August 2012 in Southwark. At the end of the study period, Lambeth had 31 full-time staff, Lewisham had 27 and Southwark had 29, reflecting their much higher than average needs for mental health treatment (North East Public Health Observatory, 2011). The teams have always operated broadly in line with the Mental Health Implementation Guide. Twenty-four hour working has been implemented through arrangements with the psychiatric liaison teams serving each of the four local Accident and Emergency departments – these can accept patients for home treatment at any time, and patients already being home treated can attend overnight if necessary. There have been some differences between the teams, for example, in the extent to which one staff member coordinates an individual's care and whether referrals are accepted directly from primary care, as in Southwark, or not, as in Croydon.

The structure of inpatient services varied over time and by borough: Lewisham operated a Triage Ward system throughout the study period, whereas Southwark operated a system in which all admissions were preferentially directed to two of its acute wards. A Triage Ward was introduced in Lambeth from the end of 2011 and in Croydon from the end of 2012, prior to which all wards were typical acute wards.

## Methods

Data came from the National Institute of Health Research (NIHR) Biomedical Research Centre (BRC) Case Register, which is an anonymised copy of the Trust's paperless electronic patient record database (Stewart *et al.* 2009), covering all care since 2006. Data management and analysis were programmed in SQL Server 2008 and Stata 12.

In an initial analysis, we extracted all periods of home treatment ending between 1st June 2008 and 31st May 2013. By joining to hospital stays, we calculated the proportions of ‘FD’ episodes (where there was home treatment during or at the end of a hospital stay) and ‘alternative to admission’ episodes (the remainder).

The other analyses were performed on a separate dataset. This main study dataset comprised all hospital stays ending with a discharge from one of the borough general psychiatric wards operated by the Trust over the same period, removing second and subsequent stays by the same individual and removing stays in which the address at discharge was outside the four boroughs. (The first restriction avoided clustering that would otherwise have complicated multiple imputation (see below); the second removed individuals ineligible for treatment by the Trust's HTTs and who could have been readmitted to other hospitals).

For each hospital stay in this dataset we extracted the following variables. Some limited recoding was performed in order to allow for small numbers in some categories and the relative difficulty of imputing multi-level categorical data:

a. whether the individual concerned had been referred for consideration of FD and the date of any such referral;
b. whether the individual was treated with FD as defined above or not;
c. the total bed days within the admission (date of discharge minus date of admission minus any nights during which overnight or extended leave was taken);
d. the date of any subsequent readmission together with any censoring events occurring in the period after discharge (moving outside the Trust catchment area or death);
e. age at admission;
f. sex;
g. ethnicity – coded as White British, Black or Other (Black Caribbean, Black British and Black Other categories were combined because of uncertainty about the interpretation of the latter category; all other ethnicity categories were too small to be analysed separately and were combined);
h. marital status;
i. having dependent children or access to children;
j. the ICD-10 primary diagnosis recorded closest to discharge;
k. the most restrictive legal status during the admission (informal; Section 2 – allowing for detention of up to 28 days; Section 3 and forensic – allowing for detention of up to 6 months or more);
l. the number of discharges from inpatient mental health services in the 2 years preceding admission (0, 1, 2, ≥3);
m. the length in days of the longest of these admissions – this was log-transformed;
n. the number of periods of home treatment starting in the 2 years preceding admission (0, 1, ≥2);
o. residential mobility (having a different address at discharge);
p. being discharged to a care home;
q. scores on individual items from the Health of the Nation Outcome Scales (HoNOS (Wing *et al.* 1998) recorded nearest to the day of admission, excluding ratings made more than 3 days prior to admission or 10 days after admission and ratings made after discharge. All item scores were recoded as ‘low’ (0–1) or ‘high’ (2–4). The other mental and behavioural problems item, which requires the rater to choose the most severe among a list of problems and rate the severity of that problem, was not used due to poor content validity, and the problems with occupation and activities item was also discarded in view of poor inter-rater reliability (Pirkis *et al.* 2005);
r. scores on individual HoNOS items recorded nearest to the day of discharge, excluding ratings made before discharge or more than 10 days after discharge, and otherwise recoded as above.

Initial descriptive and unadjusted analyses were performed. After exploring the missing data, we imputed the missing values using multiple imputation by chained equations (Van Buuren *et al.* 1999; Royston, 2004). All exposure and outcome variables used in the analyses were included in the imputation model (readmission was represented by an indicator variable for readmission and the Nelson–Aalen cumulative hazard estimator). Predictors of missingness were also included (see the Results section). Following the rule that the number of imputations should at least equal the highest percentage of missing values across all the analysis variables (White *et al.* 2011), we imputed 45 datasets. Imputed and original values were compared to check that the imputation process had produced no anomalies.

The analyses performed were as follows:

1. In order to explore the associations of being treated with FD, we performed a multiple logistic regression on the imputed datasets, combining parameter estimates from separate analyses of each imputed dataset as per Rubin's rules (Rubin, 1987). Model building followed the method suggested by Hosmer *et al.* (2008). First, the set of exposure variables (*e*)–(*r*) above were included in an initial multiple logistic regression model with receipt of FD as the outcome measure. Probability values for each variable were calculated using the Wald test. Second, all variables with *p* ≥ 0.20 were subtracted. Third, all variables with *p* > 0.05, but *p* < 0.20 were subtracted in turn, starting with the least significant, examining other coefficients for a change in value of >20%, which would indicate the possibility of confounding by the subtracted variable. Fourth, each of these subtracted variables was then retested for inclusion, aiming to re-include variables with adjusted *p* value <0.05.
2. We used the same multiply imputed data to analyse the effects of FD on readmission. Dates of readmission were transformed into survival durations relative to the day of hospital discharge. We censored observations at the time of death or when a subject moved to an address outside the catchment area of the Trust. All observations not ending in failure, death or movement outside the study area by the time of data extraction (8 November 2013) were administratively censored. A Cox regression analysis was constructed, following the same model building process outlined above.
3. We estimated the effect of FD on bed days. A linear regression model was fitted on the multiply imputed data, following the same model building process as in (1) above, and testing the use of robust standard errors to deal with heteroskedasticity (unequal distribution of residuals across values of the response variable). Because of the possibility that any association between FD and reduced bed days could either be explained by the causal mechanism of interest or by the more frequent use of FD among those who are approaching the end of a shorter admission, compared to those who are approaching the end of a longer admission – a form of reverse causality – we performed a supplementary regression analysis analysing the effect of FD on the number of days between referral for consideration of FD and discharge minus any leave nights (‘post-referral bed days’), comparing those treated with FD and those referred but not treated.

## Results

### Overall balance between admission avoidance and FD

There were 12 179 episodes of care by an HTT starting within the study period. Of these, 4351 (36%) were FDs as defined here and 7828 (64%) were not.

### Characteristics of the main study dataset

**Fig. 1.**
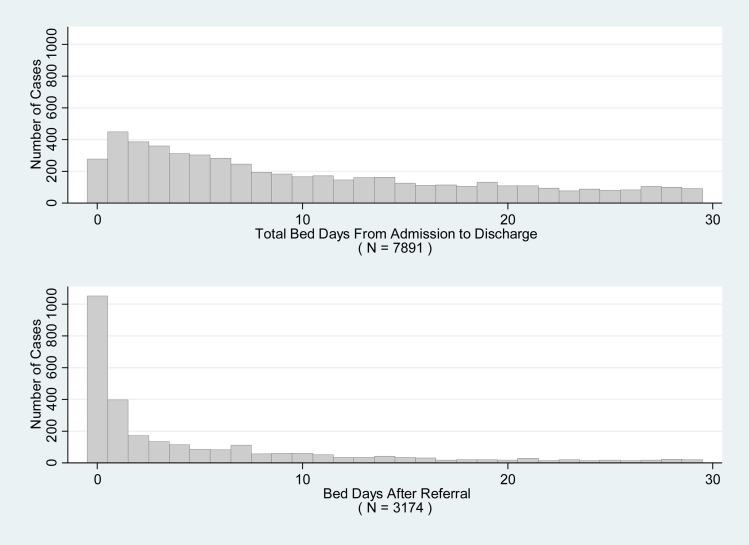
Bed days overall and post-referral bed days. *Note:* The top panel is a histogram of bed days for each admission in the main study dataset, excluding stays longer than 30 days. Bed days are calculated as discharge date minus admission date, minus any days of authorised leave during that period. The bottom panel is a histogram of bed days after referral for consideration of facilitated discharge (*post-referral bed days)*, calculated as discharge date minus date of referral for consideration of facilitated discharge, minus any days of authorised leave during that period. Again, frequencies for post-referral bed days more than 30 days are not shown. The bottom histogram only applies to the subset of individuals who were referred for consideration of facilitated discharge (*N* = 3174).

The main study dataset comprised 7891 hospital stays, of which 2274 (29%) involved an FD and 5617 (71%) did not. Overall, 41% had been referred for consideration of FD (*N* = 3174). Mean length of stay in the main study dataset was 40.3 days (s.d. 79.1 days) and the median was 18 days (interquartile range 5–46): the mean number of bed days per admission – a measure that excluded leave days and which was a main outcome of the analysis – was slightly lower, with arithmetic mean 35.1 (s.d. 63.2) and median 16. The distribution of bed days was right skewed (see Fig. 1, top panel). Eight subjects were under 18 and 165 (2%) were over 65. There were low proportions of missing data for diagnosis (3%), marital status (2%), ethnicity (2%) and residential mobility (1%). Whether or not the subject had dependent children or access to children was missing in 26% of cases, while individual HoNOS item scores at the time of admission were generally missing in about 22% of cases, and at the time of discharge in about 46% of cases. Other variables were complete. Apart from relationships with other variables, missingness for HoNOS items was related to the date of admission and ward. Overall characteristics of the sample are shown in Table 1.

**Table 1.** Characteristics of all inpatient discharges in the sample

Variable	*N* with complete data (%)	Mean (s.d.) or *N* (proportion)
Age	7891 (100%)	39.1 years (12.4)
Gender	7891 (100%)	
Male		4382 (56%)
Female		3509 (44%)
Ethnicity	7724 (98%)	
White British		3929 (51%)
Black African or Caribbean		2842 (37%)
Other		953 (12%)
Marital status	7763 (98%)	
Single		5572 (72%)
Divorced, separated or widowed		1058 (14%)
Married or cohabiting		1133 (15%)
Dependent children or access to children	5803 (74%)	
Yes		1660 (29%)
No		4143 (71%)
Primary diagnosis	7667 (97%)	
Schizophrenia (F20)		1911 (26%)
Other psychotic disorders (F21–F29)		1256 (16%)
Hypomania/mania/bipolar disorder (F30–F31)		918 (12%)
Depression (F32–F39)		1204 (16%)
Neurotic and anxiety disorders (F40–F49)		662 (9%)
Personality disorders (F60–F69)		440 (6%)
Drug & alcohol disorders (F10–F19)		873 (11%)
Other primary diagnosis		403 (5%)
Legal status^a^	7891 (100%)	
Informal		4657 (59%)
Section 2		1672 (21%)
Section 3/Forensic		1562 (20%)
Discharges ending in preceding 2 years^b^	7891 (100%)	
None		6495 (82%)
One		877 (11%)
Two		305 (4%)
Three or more		214 (3%)
Periods of home treatment ending in preceding 2 years	7891 (100%)	
None		6194 (78%)
One		1201 (15%)
Two or more		496 (6%)
Residential mobility (different address at discharge)	7891 (99%)	
Yes		680 (9%)
No		7113 (91%)
Discharged to care home	7891 (100%)	
Yes		302 (4%)
No		7589 (96%)
Non-HTT community team at discharge	7891 (100%)	
Assertive outreach		233 (3%)
Other, not assertive outreach		4903 (62%)
None		2755 (35%)
Admission HoNOS item scores		
Overactive, aggressive, disruptive or agitated behaviour	6229 (79%)	
Score 0 or 1		3803 (61%)
Score 2, 3 or 4		2426 (39%)
Non-accidental self-injury	6217 (79%)	
Score 0 or 1		4496 (72%)
Score 2, 3 or 4		1721 (28%)
Problem drinking or drug taking	6097 (77%)	
Score 0 or 1		4128 (68%)
Score 2, 3 or 4		1969 (32%)
Cognitive problems	6189 (78%)	
Score 0 or 1		4915 (79%)
Score 2, 3 or 4		1274 (21%)
Physical illness or disability problems	6182 (78%)	
Score 0 or 1		5035 (81%)
Score 2, 3 or 4		1147 (19%)
Hallucinations and delusions	6176 (78%)	
Score 0 or 1		2820 (46%)
Score 2, 3 or 4		3356 (54%)
Depressed mood	6192 (78%)	
Score 0 or 1		3036 (49%)
Score 2, 3 or 4		3156 (51%)
Problems with relationships	6100 (77%)	
Score 0 or 1		3488 (57%)
Score 2, 3 or 4		2612 (43%)
Problems with activities of daily living	6134 (77%)	
Score 0 or 1		4312 (70%)
Score 2, 3 or 4		1822 (30%)
Problems with living conditions	5873 (74%)	
Score 0 or 1		4452 (76%)
Score 2, 3 or 4		1421 (24%)
Discharge HoNOS item scores		
Overactive, aggressive, disruptive or agitated behaviour	4308 (55%)	
Score 0 or 1		3807 (88%)
Score 2, 3 or 4		501 (12%)
Non-accidental self-injury	4308 (53%)	
Score 0 or 1		3893 (90%)
Score 2, 3 or 4		415 (10%)
Problem drinking or drug taking	4295 (54%)	
Score 0 or 1		3584 (83%)
Score 2, 3 or 4		711 (17%)
Cognitive problems	4303 (54%)	
Score 0 or 1		3983 (93%)
Score 2, 3 or 4		320 (7%)
Physical illness or disability problems	4303 (55%)	
Score 0 or 1		3657 (85%)
Score 2, 3 or 4		646 (15%)
Hallucinations and delusions	4301 (55%)	
Score 0 or 1		3292 (77%)
Score 2, 3 or 4		1009 (23%)
Depressed mood	4304 (55%)	
Score 0 or 1		3276 (76%)
Score 2, 3 or 4		1028 (24%)
Problems with relationships	4288 (54%)	
Score 0 or 1		3217 (75%)
Score 2, 3 or 4		1071 (25%)
Problems with activities of daily living	4293 (54%)	
Score 0 or 1		3659 (85%)
Score 2, 3 or 4		634 (15%)
Problems with living conditions	4218 (53%)	
Score 0 or 1		3474 (82%)
Score 2, 3 or 4		744 (18%)

^a^Legal status was defined as the most restrictive section of the Mental Health Act in force during the first week of the admission. If detention was only under Section 136, Section 5(2) or Section 5(4), this was treated as informal legal status.

^b^Among those with at least one discharge from hospital in the preceding 2 years, the median length of the longest admission was 41 days (interquartile range 17–100).

Among those referred for consideration of FD, whether treated or untreated, the mean number of post-referral bed days was 17.1 days (s.d. 128.5 days). This distribution was more heavily right skewed (see Fig. 1, bottom panel), and there were many zero values: 45.6% were discharged on the same or next day.

Among those actually home treated, the mean number of days of home treatment was 27.0 (s.d. 31.6) and the median was 21 (interquartile range 11–33); the mean number of face-to-face visits was 22.8 (s.d. 23.8) and the median was 16.

### Unadjusted associations

Distributions of exposure variables, with unadjusted comparisons between those treated with FD and those who were not, are shown in Table 2. For brevity, only basic demographic variables and those variables included in the final multiple logistic regression of the odds of being treated with FD are shown.

**Table 2.** Distribution of selected variables between individuals treated with facilitated discharge (FD) and untreated individuals (non-FD)

Variable	Non-FD	FD	*p* value
Age/years	Mean 39.8 (s.d. 12.5)	Mean 38.8 (s.d. 12.3)	0.001
Gender			<0.001
Female	2369 (42%)	1140 (50%)	
Male	3248 (58%)	1134 (50%)	
Marital status			<0.001
Single	4067 (74%)	1505 (67%)	
Divorced/separated/widowed	721 (13%)	337 (15%)	
Married	729 (13%)	404 (18%)	
Ethnicity			<0.001
White British	2873 (52%)	1056 (47%)	
Black African or Caribbean	1950 (36%)	892 (40%)	
Other	661 (12%)	292 (13%)	
Diagnosis			<0.001
Schizophrenia (F20)	1300 (24%)	611 (27%)	
Other psychotic disorders (F21–F29)	787 (15%)	469 (21%)	
Hypomania/mania/bipolar disorder (F30–F31)	535 (10%)	393 (18%)	
Depression (F32–F39)	819 (15%)	385 (17%)	
Neurotic and anxiety disorders (F40–F49)	514 (9%)	148 (7%)	
Personality disorders (F60–F69)	373 (7%)	67 (3%)	
Drug & alcohol disorders (F10–F19)	774 (14%)	99 (4%)	
Other primary diagnosis	334 (6%)	69 (3%)	
Number of discharges in 2 years preceding admission			0.707
None	4615 (82%)	1880 (83%)	
One	625 (11%)	252 (11%)	
Two	217 (4%)	88 (4%)	
Three or more	160 (3%)	54 (2%)	
Longest admission ending in 2 years preceding admission			0.029
0 days or no admission	4624 (82%)	1882 (83%)	
1–17 days	249 (4%)	95 (4%)	
18–40 days	237 (4%)	100 (4%)	
41–100 days	235 (4%)	122 (5%)	
101–3323 days	272 (5%)	75 (3%)	
Number of periods of home treatment ending in the 2 years preceding admission			<0.001
None	4591 (82%)	1603 (70%)	
One	751 (13%)	450 (20%)	
Two or more	275 (5%)	221 (10%)	
Residential mobility (different address at discharge)	542 (10%)	138 (6%)	<0.001
Discharge to care home	252 (4%)	50 (2%)	<0.001
Non-HTT community team at discharge			0.332
None	1935 (34%)	820 (36%)	
Community mental health team, not assertive outreach	3519 (63%)	1384 (61%)	
Assertive outreach	163 (3%)	70 (3%)	
Admission HoNOS problem drinking or drug-taking = 2, 3 or 4	1519 (36%)	450 (24%)	<0.001
Admission HoNOS hallucinations and delusions = 2, 3 or 4	2224 (52%)	1132 (59%)	<0.001
Admission HoNOS problems with living conditions = 2, 3 or 4	1091 (27%)	330 (18%)	<0.001
Discharge HoNOS non-accidental self-injury = 2, 3 or 4	225 (8%)	190 (12%)	<0.001
Discharge HoNOS cognitive problems = 2, 3 or 4	206 (8%)	114 (7%)	0.641
Discharge HoNOS hallucinations and delusions = 2, 3 or 4	542 (20%)	467 (29%)	<0.001
Discharge HoNOS depressed mood = 2, 3 or 4	578 (21%)	450 (28%)	<0.001
Discharge HoNOS problems with relationships = 2, 3 or 4	680 (25%)	391 (25%)	0.791
Discharge HoNOS problems with living conditions = 2, 3 or 4	506 (19%)	238 (15%)	0.002

The arithmetic mean number of bed days among those treated with FD was 33.7 days; among those untreated it was 35.8 days (*t* = 1.35; *p* = 0.18). Among those referred for consideration of FD, 51.6% of those treated with FD were discharged on the same or next day *v.* 31.0% of those who were not treated with FD (chi-square *p* < 0.0001), and the mean number of bed days was 12.3 days lower in the treated group (*t* = 2.46; *p* = 0.01). The unadjusted odds ratio for discharge on the same day as referral or the next day, comparing those treated with FD to those not treated, was 2.37 [95% confidence interval (CI) 2.01–2.79; *p* < 0.0001].

In the unadjusted analysis of readmission, mean follow-up time (to failure or censoring) was 1164 days (s.d. 544 days; range 161–1985 days). The overall Kaplan–Meier estimate of the risk of readmission was 8% at 30 days, 21% at 180 days and 30% at 1 year. A log-rank test indicated that there was no unadjusted difference in rate of readmission between those who were taken on for FD and those who were not (*p* = 0.72). Comparison of imputed and original data values indicated no anomalies.

### Predictors of being an FD

The results of the multiple logistic regression of the odds of being treated with FD are shown in detail in Table 3 and are described in outline here. The only variables whose 95% CI indicated that the odds of FD were at least halved (or at least doubled) were (1) having a primary diagnosis of a personality disorder (OR 0.31; 95% CI 0.23–0.42), and (2) having a primary diagnosis of a drug and alcohol use disorder (OR 0.26; 95% CI 0.20–0.34), with both effects expressed relative to the effect of a primary diagnosis of schizophrenia. Variables associated with modestly increased odds of FD were: being married or being divorced, separated or widowed; having a diagnosis of bipolar disorder or mania (relative to schizophrenia); having been home treated within the 2 years before admission to hospital; scoring higher on the HoNOS hallucinations and delusions item either at admission or at discharge; scoring higher on the HoNOS deliberate self-harm item at discharge; and scoring higher on the HoNOS depressed mood item at discharge. Variables associated with modestly reduced odds were: male gender; having any other non-psychotic diagnosis; having a longer previous admission in the preceding 2 years; being under a community mental health team at discharge; moving to a new address during the admission; being discharged to a care home; scoring higher on the HoNOS problem drinking or drug taking item at admission; scoring higher on the HoNOS physical health problems item at discharge; scoring higher on the HoNOS problems with relationships item at discharge; and scoring higher on the HoNOS problems with living conditions item at admission or at discharge.

**Table 3.** Adjusted associations with being taken on for facilitated discharge

Variable	OR (95% CI)	*p* value
Male gender	0.86 (0.77, 0.95)	0.0052
Marital status		0.0022
Single	1	
Divorced/Separated/Widowed	1.25 (1.07, 1.45)	
Married	1.22 (1.05, 1.42)	
Diagnosis		<0.0001
Schizophrenia (F20)	1	
Other psychotic disorders (F21–F29)	1.13 (0.96, 1.32)	
Hypomania/mania/bipolar disorder (F30–F31)	1.42 (1.19, 1.70)	
Depression (F32–F39)	0.70 (0.58, 0.85)	
Neurotic and anxiety disorders (F40–F49)	0.45 (0.35, 0.57)	
Personality disorders (F60–F69)	0.31 (0.23, 0.42)	
Drug & alcohol disorders (F10–F19)	0.26 (0.20, 0.34)	
Other primary diagnosis	0.38 (0.29, 0.51)	
Longest admission with discharge from hospital in 2 years preceding current admission		0.0022
0 days	1	
1–17 days	1.01 (0.82, 1.24)	
18–40 days	0.88 (0.75, 1.03)	
41–100 days	0.79 (0.67, 0.92)	
101–3323 days	0.68 (0.55, 0.84)	
Number of periods of home treatment in 2 years before admission		<0.0001
None	1	
One	1.64 (1.43, 1.89)	
Two	2.22 (1.79, 2.76)	
Residential mobility (different address at discharge)	0.70 (0.57, 0.87)	0.0009
Discharge to care home	0.45 (0.32, 0.62)	<0.0001
Non-HTT community team at discharge		<0.0001
None	1	
Community mental health team, not assertive outreach	0.69 (0.61, 0.77)	
Assertive outreach	0.69 (0.50, 0.95)	
Admission HoNOS problem drinking or drug-taking = 2, 3 or 4	0.80 (0.65, 0.89)	0.0011
Admission HoNOS physical health problems = 2, 3 or 4	0.84 (0.72, 0.98)	0.0279
Admission HoNOS hallucinations and delusions = 2, 3 or 4	1.22 (1.07, 1.40)	0.0032
Admission HoNOS problems with living conditions = 2, 3 or 4	0.72 (0.62, 0.85)	0.0001
Discharge HoNOS non-accidental self-injury = 2, 3 or 4	1.42 (1.10, 1.83)	0.0081
Discharge HoNOS hallucinations and delusions = 2, 3 or 4	1.36 (1.15, 1.60)	0.0003
Discharge HoNOS depressed mood = 2, 3 or 4	1.51 (1.27, 1.79)	<0.0001
Discharge HoNOS problems with relationships = 2, 3 or 4	0.85 (0.72, 0.99)	0.0372
Discharge HoNOS problems with living conditions = 2, 3 or 4	0.80 (0.66, 0.98)	0.0287

### Association between FD and readmission

The lack of effect of FD on readmission observed in the unadjusted analysis was also observed in the full Cox regression model – the adjusted hazard ratio was 0.96 (95% CI 0.89–1.04; *p* = 0.32). Full results are available on request. A separate analysis of predictors of readmission based on the same dataset is forthcoming (Tulloch AD *et al.* 2014, unpublished manuscript).

### Association between FD and bed days

The coefficient for FD in the linear regression of bed days was −3.98 (95% CI −6.65 to −1.32; *p* = 0.0004). The estimated effect size was 0.06 (95% 0.02–0.11). Although examination of residuals indicated some heteroskedasticity, the very close agreement between robust and ordinary least-squares estimates of the s.e. showed that this was inconsequential. Covariates included in the model were age; diagnosis; legal status; longest admission in the 2 years preceding the index admission; residential mobility; discharge to a care home; being under the care of another community mental health team at discharge; the admission HoNOS items for cognitive impairment and problems with activities of daily living; and the discharge HoNOS items for agitated behaviour, non-accidental self-injury and physical illness. Full results of the regression are not shown but are similar to those in a previous analysis (Tulloch *et al.* 2012). *Post hoc* exploration indicated that negative confounding by diagnosis and legal status was responsible for the greater size and significance of the adjusted effect compared with the unadjusted effect: other variables did not have important confounding effects.

In the analysis of post-referral bed days among those who were actually referred for consideration of FD, the data were poorly fitted using multiple linear regression. Therefore, a generalised linear model was fitted using a gamma distribution and log-link function. The exponentiated coefficient for FD was 0.54 (95% CI 0.42–0.70); *p* < 0.0001), meaning that the adjusted arithmetic mean of the number of post-referral bed days was 46% lower among those home treated. With the data instead dichotomised, the adjusted odds ratio for discharge on the day of referral or the next day among those home treated was 2.69 (95% CI 2.27–3.20; *p* < 0.0001). In both the analyses, the least-squares and robust estimates of the s.e. agreed very closely, indicating that any remaining heteroskedasticity was inconsequential.

## Discussion

### Summary and discussion of main findings

The episodes of home treatment that we characterised as FD comprise 36% of the total activity of HTTs in the NHS Trusts from which we took our data and FD was used in 29% of admissions. Therefore, while FD constitutes a significant part of the workload of HTTs, many service users continue to be discharged without the use of home treatment.

Through the use of demographic, clinical and service use data recorded in an electronic patient record, we found a number of modest-sized associations with the odds of being treated with FD. We assume that HTT members routinely make judgements regarding potential clients’ suitability for home treatment at the point of referral: our results, taken together, may help to illustrate some aspects of this selection process, although there are some important factors that we were unable to directly measure (in particular, a person's willingness to be home treated – discussed below).

The positive associations that we found with having a primary diagnosis of a psychotic mental illness and with the HoNOS item scores for self-harm, hallucinations and delusion and depressed mood are consistent with HTTs continuing to treat those with severe functional mental disorders (Department of Health, 2001). The converse applies to the negative associations with primary diagnosis of non-psychotic illness, with the HoNOS item score for drug and alcohol problems and with the HoNOS cognitive problems item. These findings, which include the largest effects that we observed, are consistent with those of the studies summarised by Cotton *et al.* (2007).

Many of the other associations that we found, although modest in size, may reflect the importance of more practical considerations. For example, the reduced odds of treatment when someone is already under the care of a Community Mental Health Teams or will reside in a carehome may reflect a judgement that at least some of such individuals will be adequately served without additional support. The lower chance of being taken on for FD among those who moved home during the admission or at the point of discharge and among those who were rated as having problems with living conditions, support our experience that HTTs regard stable living arrangements as a prerequisite for home treatment. The availability of family members to support the delivery of care may similarly explain the modest positive associations seen with being married, divorced, separated or widowed rather than single and the negative association with the HoNOS problems with relationships item.

It is less easy to explain the association with gender, which was not accounted for by having dependent children or access to children, contrary to one previous study (Dean & Gadd, 1990). One possibility is that female service users are more receptive to the prospect of home treatment. Alternatively, it may be that male patients are more often unsuitable because of their risk history, which we did not measure directly. Similarly, the lower odds of treatment among those who had had a longer hospital admission in the preceding 2 years is perhaps most likely to be explained by selection on some other, unmeasured variable, given that HTTs would be expected to treat a group of individuals more at risk of protracted admission (Tulloch *et al.* 2012).

The postulated decision-making process around referrals can directly be invoked to make sense of the modest, dose-dependent, association between FD and previous home treatment: if previous home treatment reduces the uncertainty in the mind of clinicians about the likely suitability of a potential client, this may make treatment more likely to be offered.

Our analysis of bed days suggests that FD produced a reduction in LOS of 4 days. This reduction was small in relation to overall variation in bed days, with an estimated Cohen's d of 0.06. The same direction and statistical significance of effect was seen when the sample was restricted to those referred for HTT and the number of bed days was instead calculated between the day of referral and the day of discharge, supporting the interpretation of the main estimate as a small causal effect, uncontaminated by reverse causal effects. However, the rate of readmission did not differ between those who treated with FD and those who were not. Taking these three analyses together we suggest that FD, as practiced in our sample, very modestly reduced bed use, and did so without adverse (or beneficial) effects on readmission. These are more positive findings than have been obtained in some other studies of home treatment (see introduction above). Even a reduction of 4 days in the length of an admission may be worthwhile from the point of view of providing humane and acceptable care, although it should be borne in mind that other service costs might very well outweigh the reduced costs attributable to this effect. Of note, we found that those treated received, on average, 22 face-to-face visits, constituting a significant substitution of community for inpatient care.

### Strengths and limitations

Strengths of the study are its being the first study to specifically examine the extent to which FD is used, its associations and its outcomes; the large sample of individual patient-level data; and the large number of clinical and service use variables that we were able to use.

One limitation of our study is that data derive only from a single NHS Trust serving predominantly inner city neighbourhoods (Lambeth, Lewisham and Southwark) or a mixture of inner city and suburban neighbourhoods (Croydon), and for much of the study period, services were provided by only four multidisciplinary teams. Our findings do not exclude the possibility that other NHS Trusts and other teams may have developed services that have different patterns of use and different effects on service use. Certainly, if area level factors or the overall level of psychiatric morbidity have any impact on treatment effectiveness, then we might expect some significant differences from effects seen elsewhere in non inner-city metropolitan areas (Glover *et al.* 1998; North East Public Health Observatory, 2011).

Our analyses were also based on observational data taken from electronic health records collected in routine practice. Most demographic, clinical and service use variables were complete or near complete, but missing data affected some other variables – especially discharge HoNOS scores – and, although we used the best available techniques to accommodate these missing values, the estimates of association between the affected variables and the odds of FD should be viewed cautiously. More generally, it may be objected that the reliability and validity of the measures that we used are unknown and possibly dubious. While we acknowledge that diagnosis (for example) may not have been measured to ‘research standards’, and HoNOS item scores may be imperfect measures of the related symptoms and behaviours, it seems to us most unlikely that this would have systematically biased our results.

The greatest limitation of our analyses is our reliance on observational data to estimate causal effects. We attempted to address the risk of unmeasured confounding by the inclusion of a broad range of clinical and demographic covariates, but as with most treatments or interventions, there may have been some selection bias: those who were thought to be more likely to respond to home treatment – and this includes those who simply agreed to be treated, rather than refusing – may have been preferentially selected both by the referring wards and the assessing HTT. However, some of those who were not selected for home treatment may have been perceived as too well to require it, and to the extent that this was not accounted for in the discharge HoNOS ratings, this would conceivably have reduced the apparent effect of FD.

A particular problem in the case of the main analysis of bed days is the possibility of reverse causality. Our supplementary analysis looking at bed days subsequent to referral among those who were referred for consideration of FD is not susceptible to the same problem and provides support for the main analysis. A better means of avoiding bias due to selection and reverse causality would have been to perform an instrumental variable analysis (Angrist & Pischke, 2009, p. 117; 151–154) but this was not possible with the available data.

### Implications for future research

We believe that our findings deserve further exploration and attempts at replication, preferably using complementary non-experimental or experimental methods, and studying other settings. Certainly, we hope to increase the extent to which FD is seen as a centrally important part of the practice of HTTs, and to provoke interest in discovering more about this work and about variation in practice. Our analysis does not allow us to determine the effect of FD on treatment costs overall – a point noted above – and we hope to extend our analysis to cover this, entailing consideration of inpatient costs, HTT costs and costs of other community teams. There may, in addition, be other potential benefits to FD that we did not measure and which would merit further attention. In particular, the finding that suicide among inpatients appears to have declined as areas have introduced HTTs (While *et al.* 2012), although potentially due to shifting of suicide from hospital to community, could at least in principle be explained by an effect of home treatment on patients who, while they remain inpatients for administrative purposes, have been sent on leave under the care of a HTT.
